# Delayed Traumatic Intracerebral Hematoma: A Pathophysiological Classification and Literature Review

**DOI:** 10.7759/cureus.42987

**Published:** 2023-08-05

**Authors:** Robert Ziechmann, Sami M Pathak, Jonathan Welch, Philip Villanueva

**Affiliations:** 1 Neurosurgery, Temple University Hospital, Philadelphia, USA

**Keywords:** delayed traumatic intracerebral hematoma, delayed post traumatic hemorrhage, traumatic brain injury, neurotrauma, neurocritical care, intracerebral hematoma, decompressive hemicraniectomy, de novo hematoma, gcs

## Abstract

Delayed traumatic intracerebral hematoma (DTICH) is a relatively common occurrence after a traumatic brain injury (TBI). Several case series have been performed to study DTICH, many of which offer different definitions of DTICH. Some definitions involve a delayed progression of an existing hemorrhage, and others involve a de novo intracerebral hematoma that was not evident on the initial trauma evaluation. We propose a classification system for DTICH that accounts for the subtleties in the clinical manifestation and pathophysiology of the different types of DTICH, with the ultimate goal of providing strategies to prevent and manage DTICH.

Based on the senior author's clinical experience, we generated a classification system for DTICH, and each type of DTICH was illustrated with a case. We defined type 1A (case 1A), the classic presentation of DTICH as predominantly characterized in the literature, as an intracerebral hematoma unseen on initial computed tomography imaging that typically develops five days to one week following blunt or penetrating head trauma. We defined type 1B (case 1B) as a hematoma that forms after at least one week following trauma in areas of the brain initially hemorrhage-free. We defined type 2 (case 2) as a hematoma that develops rapidly following a surgical evacuation of a different hematoma. We defined type 3 (case 3) as a hematoma that develops after a traumatic head injury in areas of non-hemorrhagic contusion, usually frontal or temporal.

A literature review was performed using select terms on PubMed to find articles related to DTICH, excluding articles describing DTICH from an underlying vascular injury. After performing the literature review and screening articles by title and/or abstract, a total of 79 articles were found to meet the inclusion and exclusion criteria. We recorded which type of DTICH from our classification system best correlated with the articles in our literature review.

Taken together with results from the literature, the proposed classification system is based on the senior author’s clinical experience. Overall, DTICH is a relatively common occurrence after head trauma, and our pathophysiologic classification has the potential to help outline future studies to recognize and prevent the development of DTICH.

## Introduction and background

Delayed traumatic intracerebral hematoma (DTICH), also referred to as delayed post-traumatic hemorrhage (DPTH), has been described as a sequela of a traumatic brain injury (TBI) since 1891. Bollinger et al. [1] originally reported the clinical phenomenon, termed “traumatische spät Apoplexie” (late traumatic apoplexy), in a series of four cases, in which intracerebral hemorrhage associated with acute neurologic decline occurred several days after an asymptomatic interval following a head trauma. This definition excluded cases in which there was an underlying vascular abnormality. In the era of modern computed tomography (CT), numerous case series and reports have shown this phenomenon to be the manifestation of a de novo intracerebral hematoma not present on the initial trauma evaluation [2-4]. The phenomenon has primarily been described in blunt head trauma [5] but occurs following penetrating trauma as well.

DTICH is a relatively common phenomenon, with a reported incidence of up to 7% of head trauma [6]. Several past series have classified and narrowed the definition of DTICH. Fukamachi et al. [4] developed a classification system for traumatic intracerebral hematomas based on CT scans of patients experiencing traumatic head injuries. The classification consisted of four types of traumatic intracerebral hematomas: type I involved intracerebral hematomas that were present on the initial CT scan and may have decreased in size following the initial scan; type II involved hematomas that began as small or medium size on the initial CT scan but became larger on subsequent CT scans; type III referred to hematomas that did not show alterations on the admission CT scan; type IV involved hematomas with a “salt and pepper” or “flecked high-density” (or focal high-density imaging finding) appearance on the initial CT scan [4]. In particular, Fukamachi et al. [4] defined DTICH as a hematoma that is not seen on the initial CT but does appear on subsequent CT. Alvarez-Sabin et al. [5] required that a CT scan within six hours of admission be normal for the definition of DTICH. In particular, Alvarez-Sabin et al. [5] identified 10 patients (4.5%, from a series of 216 patients with intracerebral hemorrhage) that had DPTH according to their criteria: normal head CT scans collected within six hours of the initial head injury, absence of an underlying vascular disease, neurological abnormalities following a length of time with no symptoms, and intracerebral hemorrhage evident on the following head CT scan.

The classic clinical picture described by Bollinger et al. [1] and subsequent authors is not the only pattern of DTICH. The formation of a de novo contralateral hematoma following decompressive hemicraniectomy is also common and can be devastating [7]. Less commonly, de novo hematomas can form in an area of the brain that would otherwise seem uninjured on the head CT scan [8].

In this paper, we propose a classification system for DTICH that accounts for these clinical phenomena and their underlying pathophysiology. The ultimate goal of this research is to offer strategies for both the prevention and management of DTICH.

## Review

Methods

A retrospective review of cases was performed to illustrate each type of DTICH. The patients were adults treated at our institution, which sees a high volume of patients with traumatic injuries, from 2017-2022. In particular, we analyzed the cases and then de-identified them according to standard practices. The inclusion criteria for the cases required that the initial traumatic injury of the patient be one of the following: subdural hematoma, epidural hematoma, cerebral contusion, traumatic subarachnoid hemorrhage, or skull fracture. Another inclusion criterion was the presence of the delayed formation of an intracerebral hematoma after the patient’s initial injury. For the exclusion criteria, we did not include any patients who had an underlying intracranial vascular injury as identified by computed tomography angiography (CTA) or digital subtraction angiography.

A comprehensive literature review was performed to evaluate previous articles that discuss DTICH. All literature searches were made in 2020. We searched the following terms: “delayed,” “traumatic,” “intracerebral” (or “intracranial”), and “hematoma” (or “hemorrhage”) on PubMed. In addition, a separate review was performed using the terms “vascular,” “pathophysiology,” “traumatic,” “brain,” and “injury” on PubMed. Articles were screened by the title and/or abstract for relevance to the topic of DTICH and included accordingly. Included were case reports, case series, literature reviews, and original research relating to DTICH. Excluded were articles describing delayed traumatic intracerebral or intracranial hematoma or hemorrhage from an underlying vascular injury. For each article that met the inclusion and exclusion criteria in our literature review, we recorded the year of publication, authors, number of cases of DTICH, time to discovery of DTICH, incidence of DTICH, clinical presentation, means of diagnosis of DTICH, method of evaluating for vascular lesion, location of DTICH, and type of DTICH based on our proposed classification system. Several of the articles did not include all of these pieces of information, so we recorded all of the information available. Based on our senior author’s clinical experience and knowledge of the literature, patterns of clinical presentation, and the information obtained from the literature, we generated a classification system to characterize the different types of DTICH.

Knowledge obtained from the literature was integrated with the retrospective review of the cases to further characterize each type of DTICH. For each type, we provide the de-identified case description, clinical definition, pathophysiology, and expected imaging findings.

Classification system and illustrative cases

The classification system we propose describes three distinct entities in terms of clinical presentation and pathophysiology. The first type is subdivided due to subtle but important differences, primarily in the clinical presentation.

Case 1A (DTICH type 1A)

**Figure 1:**
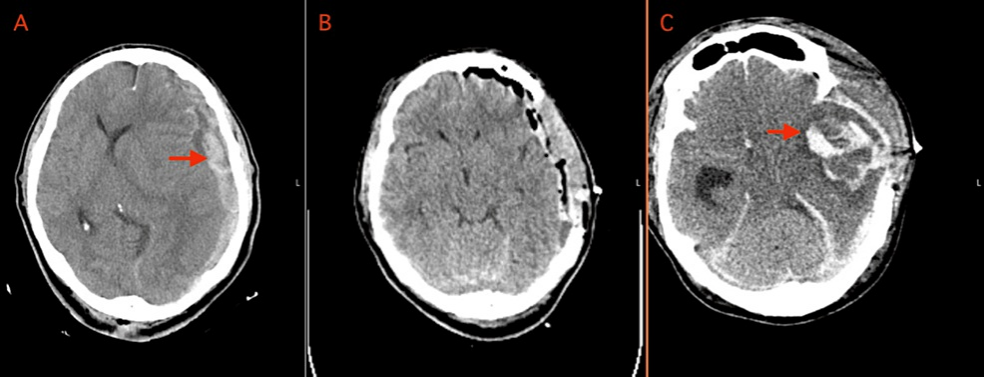
CT images demonstrating DTICH type 1A The left image (panel A) is the initial CT scan on POD 0 for a patient who eventually developed DTICH type 1A. The patient was found to have a massive left-sided acute subdural hematoma (red arrow in panel A) with 2 cm midline shift on the initial trauma evaluation. The middle image (panel B) is a CT scan for the same patient on POD 1 after undergoing left craniectomy. The right image (panel C), a CT scan for the same patient on POD 7, shows a large intracerebral hemorrhage (red arrow in panel C) with effacement of the basal cisterns. CT: computerized tomography; POD: post-operative day; DTICH: delayed traumatic intracerebral hematoma

Case illustration: The patient is an approximately 50-to-60-year-old man who initially presented with a rapid neurologic decline after being struck by a motor vehicle. On the initial exam, he had a Glasgow Coma Scale (GCS) score of 3, bilaterally non-reactive pupils, and absent brainstem reflexes. On trauma evaluation, he was found to have a massive left-sided acute subdural hematoma with 2 cm shift (Figure 1). He had subsequent improvement in his exam to the point of extensor posturing, and the family was offered surgical intervention with the caveat of a poor neurologic prognosis. He underwent left craniectomy, and his exam improved to a GCS score of 8 with eyes (E), motor (M), and verbal (V) scores of 2, 5, and 1 (E2M5V1), respectively. An intracranial pressure (ICP) monitor was placed on post-operative day (POD) 2 given the GCS score. The monitor demonstrated an ICP below 20 mmHg and was subsequently removed. His exam remained overall stable until an acute decline on POD 7 when he had a GCS score of 3 and fixed and dilated pupils. The head CT scan showed a large intracerebral hemorrhage with effacement of the basal cisterns (Figure 1). A goals-of-care discussion was conducted with the family who decided not to escalate care given a poor prognosis overall, and the patient passed away.

Definition: Type 1A is the classic presentation of DTICH, as predominantly described in the literature. We define type 1A as an intracerebral hematoma, unseen on initial head CT imaging, that develops typically five days to one week following blunt or penetrating head trauma. The hematoma may develop in areas of prior visualized encephalomalacia. Cases with underlying vascular lesions seen on angiography are excluded. DTICH type 1A is often heralded by an acute change in GCS score, seizure, or new focal neurologic deficit.

Pathophysiology: In the initial description, Bollinger et al. [1] postulated that delayed hematoma formed in areas of focal parenchymal softening and vascular dysfunction. Subsequent studies have supported this theory, summarized well by Fukamachi et al. [4]. Studies have reported a peak incidence of five days to one week following an injury and a common final pathway of vascular fragility leaking to an eventual microvascular rupture. Five days to one week following injury is the peak time for blood macrophage infiltration of inflamed tissues, promoting a perivascular leak and enzymatic and free radical injury of the vessel wall [9,10]. Blood-brain barrier (BBB) disruption at the capillary level peaks at this time due not only to a disruption in tight junctions but also to an inflammation-mediated upregulation in transendothelial transport mechanisms [11]. In addition, post-traumatic vasospasm can occur during this time period and is associated with decreased cerebral blood flow and worse outcomes [12]. The parenchymal softening, or encephalomalacia, that Bollinger et al. [1] described likely creates a potential space for the hematoma to form, whether it be by a gradual accumulation of the vascular leak or by an acute microvascular rupture.

Imaging: The initial, non-contrast CT imaging is negative for hematoma in the area that will develop hematoma. Hypodensity may be seen, especially in the frontal or temporal lobes and at the gray-white junction. This hypodensity reflects the ongoing development of the parenchymal softening and perivascular leak, which may be better reflected on magnetic resonance imaging (MRI) where an increased T2-weighted signal can be seen where DTICH will occur [13].

Case 1B (DTICH type 1B)

**Figure 2:**
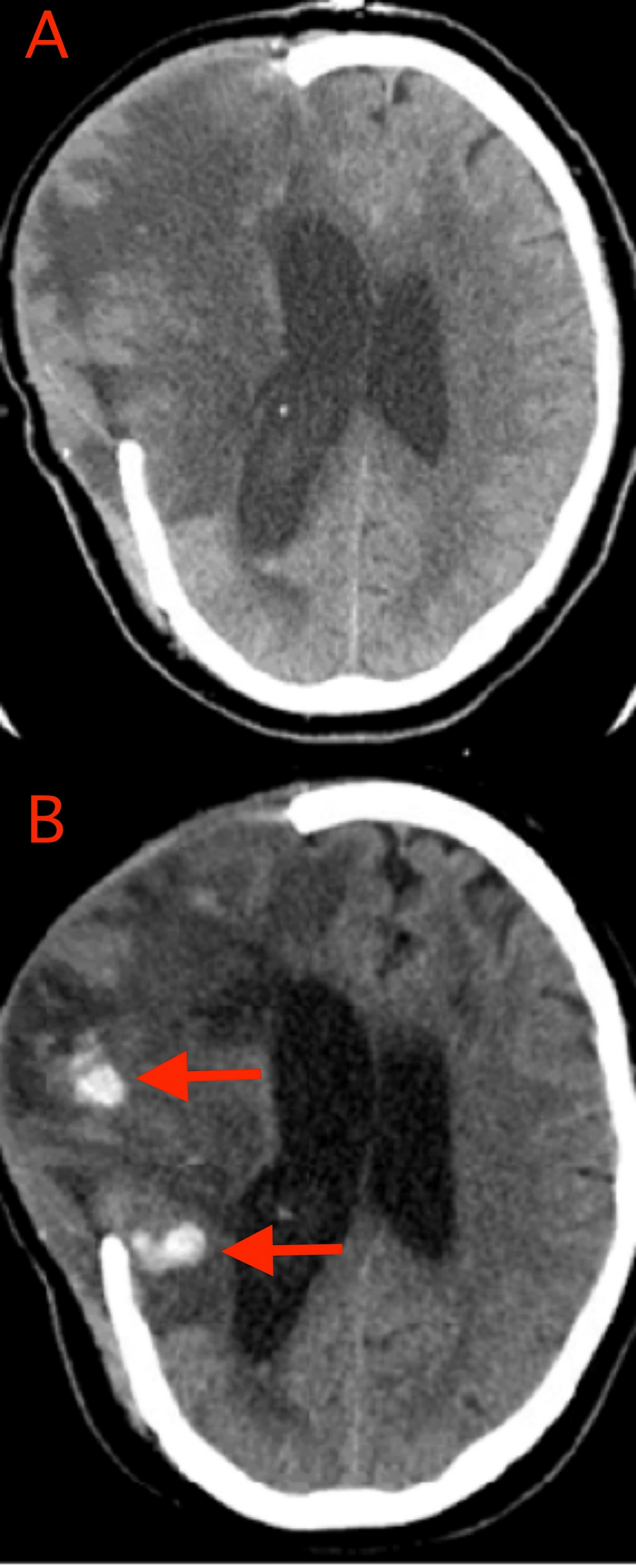
CT images demonstrating DTICH type 1B CT images on POD 22 (top panel A) and 25 (bottom panel B) for a patient experiencing DTICH type 1B are shown. The bottom image shows small hematomas within the frontal and temporal lobes (red arrows in panel B) not seen on imaging three days prior. CT: computerized tomography; POD: post-operative day; DTICH: delayed traumatic intracerebral hematoma

Case Illustration: The patient is a man of approximately 40 to 50 years of age who presented after a gunshot wound to the head. On the initial exam, he had a GCS score of 7 (E1M5V1), localizing on the right and withdrawing with non-antigravity strength on the left. The head CT scan showed a bullet trajectory through the right frontal lobe, right temporal lobe, and right cerebellum. The CTA of the head at the time of injury (and on a delayed basis) showed no vascular abnormality. The patient underwent right hemicraniectomy and external ventriculostomy drain (EVD) placement. Post-operatively, his GCS score was 9 (E4M4V1). On POD 25, the head CT scan showed small hematomas within the frontal and temporal lobes not seen on imaging three days prior (Figure 2). There was no associated change in the GCS score or ICP. Deep vein thrombosis prophylaxis was performed. The hematomas were monitored with serial imaging and were stable. The patient had a prolonged hospital course but was discharged to a nursing facility two months later with a GCS score of 10 (E4M5V1, tracheostomy).

Definition: We define type 1B DTICH as hematomas that form more than one week following trauma in areas of the brain initially free of hemorrhage. The hematomas form in the acute to sub-acute post-injury phase. These delayed hematomas may be seen in patients with a low GCS score during routine follow-up imaging scans. There may be associated elevated ICP. The clinical picture often does not change significantly (i.e., presents with a lack of improvement). Hematomas may occur in the context of encephalomalacia.

Pathophysiology: Similar to type 1A, the etiology of type 1B is likely hematoma formation in areas of encephalomalacia due to a vascular leak and microvascular rupture [9,10]. The difference in timing for these long-delayed hemorrhages is also believed to be due to a neuroinflammation process. In this case, microhemorrhages lead to the deposition of hemosiderin, which has cytotoxic effects that can lead to an inflammatory cascade and the recruitment of macrophages [14]. These smoldering neuroinflammatory changes are also thought to underlie epileptogenesis in patients with TBI [11]. In addition to disruption of the BBB, long-term changes post-TBI lead to a relative increase in the resting cerebral blood flow in injured areas as seen on perfusion imaging [15]. This is likely due to both angiogenesis and vasculogenesis, which are known to be induced by macrophages [16]. Perfusion studies also show injured areas in patients with chronic TBI to have impaired cerebrovascular reactivity [17]. We propose that in this setting, an otherwise relatively benign event, such as a subclinical seizure, would be enough to cause changes in the blood flow and blood pressure to cause hemorrhage in vessels rendered fragile by chronic inflammatory changes.

Imaging: Repeat imaging in patients with head trauma will demonstrate hematomas in an area of the brain that was negative for hematomas on the initial non-contrast head CT. The hematoma of type 1B is generally associated with hypodensity representing encephalomalacia [18].

Case 2 (DTICH type 2)

**Figure 3:**
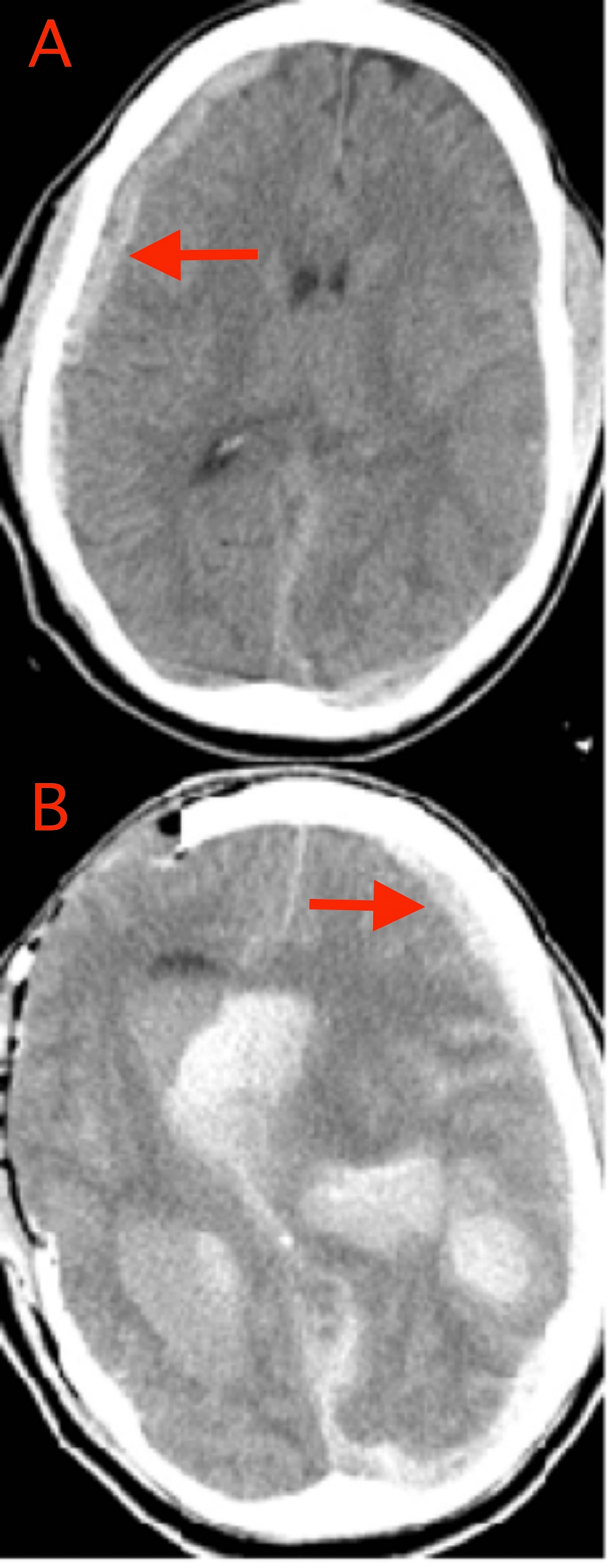
CT images demonstrating DTICH type 2 CT images for a patient experiencing DTICH type 2 are shown. The top image (panel A) represents the pre-operative CT scan, which demonstrated a right-sided subdural hematoma (red arrow in panel A). The patient underwent a right hemicraniectomy and evacuation of the right-sided subdural hematoma, and the bottom image (panel B) represents the postoperative CT scan performed immediately following surgery. The bottom image (panel B) shows a new left-sided intracerebral hematoma, intraventricular hemorrhage, and left-sided subdural hematoma (red arrow in panel B). CT: computerized tomography; DTICH: delayed traumatic intracerebral hematoma

Case Illustration: The patient is an approximately 40-to-50-year-old man who was struck by a motor vehicle. On presentation, his GCS score was 4 with a fixed and dilated right pupil. Mannitol was given en route to the operating room, and he was localizing as the best motor exam before surgery. He underwent a right hemicraniectomy and evacuation of a right-sided subdural hematoma. Post-operative head CT (Figure 3) was done immediately following surgery as a routine scan showed a new left-sided intracerebral hematoma, intraventricular hemorrhage, and left subdural hematoma, for which the patient was taken immediately back to the operating room for left hemicraniectomy.

Definition: DTICH type 2 is typically a postoperative phenomenon, which manifests rapidly following the evacuation of a different hematoma. A new intracerebral hematoma forms in an area of the brain that was normal on the initial CT scan, typically contralateral to the initial hematoma. There is no underlying vascular lesion on angiography. The hematoma often forms within 24 to 48 hours of the initial decompressive craniectomy. However, DTICH type 2 may occur more rapidly and be seen on initial postoperative imaging or even intraoperative imaging.

Pathophysiology: The formation of a hematoma likely reflects de-tamponading of potential bleeding diathesis by the removal of the initial hematoma or decompressing bone. With the removal of the initial mass lesion, lowered ICP permits new, delayed hemorrhage, primarily through venous oozing into the areas of cerebral contusion [19]. The initial insult may be an acute shearing of microvessels, similar to the vessel injury in herniation that causes Duret hemorrhages.

Imaging: Initial CT imaging will demonstrate a hematoma (epidural, subdural, or intraparenchymal), but no hemorrhage in the future area of DTICH type 2. However, following surgical evacuation, repeat imaging will demonstrate new hemorrhages, often contralateral to the initial hematoma. Furthermore, there may be associated contralateral calvarial fracture or pneumocephalus.

Case 3 (DTICH type 3)

**Figure 4:**
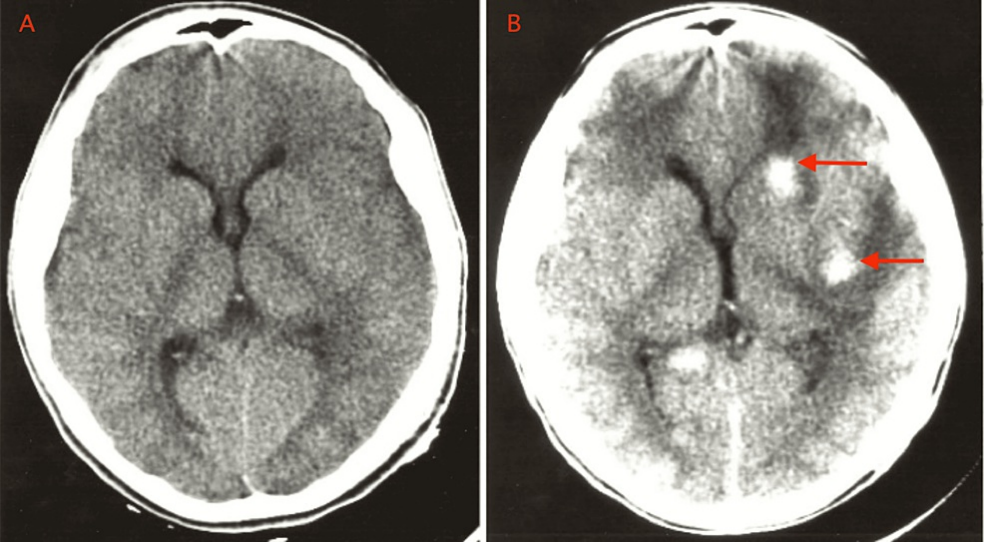
CT images for a patient with DTICH type 3 CT images for a patient experiencing DTICH type 3 are demonstrated. The left image (panel A) shows the initially negative CT scan. The patient had other traumatic injuries and was taken to the operating room. The right image (panel B) demonstrates the resulting CT scan following post-operative neurologic changes. The patient was found to have de novo hematoma (red arrows in panel B) in the follow-up head CT. CT: computed tomography; DTICH: delayed traumatic intracerebral hematoma

Case illustration: The patient is an approximately 20-to-30-year-old man with an unclear mechanism of trauma. He had an initial GCS score of 14, and the head CT scan reads as negative. On hospital day 1, the patient underwent exploratory laparotomy and open reduction and internal fixation of a tibia fracture. He had wide variability in his blood pressure measurements in the operating room. He developed right hemiparesis in the post-anesthesia care unit, prompting head CT acquisition. Initial and postoperative head CTs are shown in Figure 4. The patient developed de novo hematoma after the initial head CT scan was read as negative. The patient received an ICP monitor and underwent medical management.

Definition: A hematoma forms after a traumatic head injury in areas of non-hemorrhagic contusion, usually frontal or temporal. A hematoma typically forms after intraoperative hypotension and rebound hypertension, hypoxia, hypercarbia, blood loss, or rises in intraabdominal or intrathoracic pressure [4,8]. Oftentimes, the patient undergoes surgery for non-neurological trauma injury. Hypotension occurs secondary to blood loss from other sustained injuries. Following surgery, the patient presents with a worsened neurologic status.

Pathophysiology: Following a traumatic head injury, brain contusion may cause disturbed autoregulation, vasoreactivity, and vascular permeability [20]. Further dysregulation or altered physiologic parameters can cause hemorrhage into the contused area of the brain. In the setting of hypotension, subsequent large-volume resuscitation can overwhelm the impaired vasculature, and the increased intravascular pressure may lead to hemorrhage [8]. Hypoxia may lead to changes that increase vascular permeability and risk of hemorrhage [20]. Hypercarbia leads to the local vasodilation of the cerebral vasculature that can result in perivascular hemorrhage [8].

Imaging: Initial CT imaging may show intraparenchymal contusion without hemorrhage. Repeat imaging following a further physiologic insult will show intraparenchymal hematomas in the area of the initial contusion.

Literature review

A literature review was performed using PubMed and the terms “delayed,” “traumatic,” "intracerebral" (or “intracranial”), and “hematoma” (or “hemorrhage”). This yielded a total of 846 peer-reviewed articles. A separate literature review was performed using PubMed and the terms “vascular,” “pathophysiology,” “traumatic,” “brain,” and “injury.” This yielded a total of 791 peer-reviewed articles. After the articles were screened by title and/or abstract, a total of 79 articles met the inclusion and exclusion criteria for DTICH and its underlying pathophysiologic mechanisms, respectively. The most common reasons for exclusion were articles involving cases without a new intracerebral hematoma (rather an extension or worsening of an existing hematoma) and cases involving a vascular lesion underlying the intracerebral hematoma.

Articles that we added to our discussion were cited. Others were not cited due to redundancy. The case series describing DTICH that we cited are listed in Table 1. The series ranged in case numbers from two to 42 patients. The incidence ranged from 0.2% to 9.5%. The reasons for the evaluation of hematoma varied, including headache, focal deficit, seizure, altered mental status, and acute herniation syndromes. The time to presentation varied widely, anywhere from one hour to 120 days; this variability is further addressed in the description of the DTICH subtypes. Across all the series, the treatment with operative and/or medical intervention was performed according to the treating surgeon’s judgment.

**Table 1:** Chronological list and descriptions of series reporting delayed intracerebral hematoma DTICH: delayed traumatic intracerebral hematoma; n/a: not applicable; CT: computed tomography; MRI: magnetic resonance imaging

Year	Author(s)	Number of Cases	Time to Discovery	Incidence	Presentation	Means of Diagnosis	Evaluation for Vascular Lesion	Location of DTICH	Type(s) of DTICH
1891	Bollinger et al. [1]	4	3 days-3 weeks	n/a	Hemiplegia, coma, seizure	Carotid angiography, necropsy	Carotid angiography	n/a	Type 1A
1970	Morin and Pitts [21]	20	1-120 days	n/a	n/a	Carotid angiography, ventriculogram, surgery, necropsy	Carotid angiography	n/a	n/a
1972	Baratham and Dennyson [2]	21	5 hours– 14 days	21/7866 (0.2%)	n/a	Carotid angiography, echoencephalography	Carotid angiography, necropsy	Frontal (11), temporal (8), parietal (3), basal ganglia (2), cerebellar (2)	Types 1A, 1B
1978	Brown et al. [22]	2	5-10 hours	n/a	Unresponsive, fixed/dilated pupil	Head CT	None	Frontal (1), temporal (1)	Types 1A, 2
1979	Gudeman et al. [23]	12	24 hours-24 days	12/162 (7.4%)	none	Head CT	None	Frontal (3), occipital (1), temporal (4), parietal (1), multiple (3)	Types 1A, 1B, 2
1979	Hirsh [19]	4	7 hours-8 days	n/a	Dilated pupil, headache, poor neurologic status	Head CT	None	Frontal, temporal, occipital	Type 2
1984	Ninchoji et al. [24]	25	8 hours-7 days	25/775 (3.2%)	Coma, focal deficit, seizure	Head CT	None	n/a	Types 1A, 2
1984	Young et al. [25]	15	8 hours-4 days	n/a	Hemiparesis, coma, intracranial pressure rise	Head CT	None	Frontal (9), cerebellar (1), temporal (5)	Type 1A
1984	Sawada et al. [6]	18	5 days	18/268 (6.7%)	n/a	Head CT	None	n/a	Type 1A
1985	Fukamachi et al. [4]	42	5 hours-7 days	n/a	n/a	Head CT	Catheter angiography	n/a	Types 1A, 2
1986	Atluru et al. [18]	5	3 days-3 weeks	n/a	Headache (2), seizure, hemiparesis	Head CT	Catheter angiography (1)	Temporal (2), parietal (2), midbrain	Types 1A, 1B
1988	Tanaka et al. [13]	4	8-25 hours	4/42 (9.5%)		Head CT, brain MRI	None	Frontal, temporal	Type 1A
1990	Elsner et al. [3]	2	10 - 14 days	n/a	Coma (2)	Head CT	None	Frontal (1), parietal (1)	Type 1B
1991	Lee and Lui [8]	3	4-12 hours	n/a	Coma	Head CT	None	Frontal (3)	Type 3
1995	Álvarez-Sabín et al. [5]	10	1-15 days	10/216 (4.6%)	Hemiparesis (7), cranial nerve palsy (1), aphasia (1), pain (1)	Head CT	Catheter angiography	Basal ganglia (6), frontal (3), pontine (1)	Types 1A, 1B
2003	Kaplan et al. [26]	2	92-168 days	n/a	Headache, dysarthria, hyperkinesia	Head CT	Catheter angiography	Temporal (2)	Type 1B
2008	Flint et al. [27]	23	n/a	n/a	n/a	Head CT	None	n/a	Type 2
2008	Yang et al. [7]	8	1-7 hours	8/108 (7.4%)	n/a	Head CT	None	n/a	Type 2

Discussion

DTICH, originally described by Bollinger et al. [1] in 1891, is the development of a delayed de novo hematoma after a traumatic head injury. DTICH was the focus of significant studies during the 1970s and 1980s. With the advent of CT, DTICH was characterized as a hematoma formation in the setting of an initially normal CT scan [5]. The majority of series of DTICH were reported during that time period, and in recent years, there have been fewer literature works on the subject. However, DTICH is not an uncommon phenomenon, with a reported incidence of up to 7% of head trauma [6]. The drop in reports on the subject likely represents a loss of recognition in neurosurgery regarding this phenomenon. A renewed focus on DTICH is warranted, as the phenomenon is associated with high morbidity and mortality and requires elevated critical care [28].

Some of the past descriptions and definitions of DTICH do not account for other etiologic phenomena reported in the literature, including, for example, contralateral hematoma development after craniectomy [7] and hematomas that develop in areas of seemingly unaffected brain [8]. The classification system of Fukamachi et al. [4] focuses on the characteristics of the initial hematoma and the transformation of the initial imaging findings. Our classification system is different in that we distinguish the type of DTICH based on the time interval for the development of DTICH after an injury (more than or less than one week, as in type 1A versus 1B) and by injury mechanism, for instance, following decompressive craniectomy for unrelated post-traumatic hematoma or following physiologic derangement in areas of non-hemorrhagic contusion in types 2 and 3, respectively. Not every TBI is the same, and each of the distinct etiologies and presentations of DTICH requires a nuanced approach. In the current report, we propose a classification system, based on available literature and our senior author’s 40-year clinical experience, that encapsulates the distinct pathophysiologic mechanisms responsible for DTICH formation. We separated DTICH into types 1A, 1B, 2, and 3.

We defined DTICH type 1A as hematomas that develop within one week following a TBI in areas initially free of hemorrhage on CT imaging. Type 1A may occur after trauma due to a vascular leak and microvascular rupture with secondary hemorrhage coalescence in areas of encephalomalacia [9,10]. Type 1A is the classic description of delayed hematoma and is the predominant form of DTICH reported in the literature. Type 1A is a highly morbid phenomenon [2] and, in our experience, requires surgical decompression of the hematoma. However, strategies for the prevention and early identification of type 1A are not solidified. Type 1A is associated with increased signals on T2-weighted MRI [13], so early MRI for patients with traumatic head injuries may be advisable. The identification of additional imaging changes predictive of type 1A formation will allow for screening and early adjustments in management.

DTICH type 1B is the formation of hematoma more than one week after head trauma in areas of the brain without hematoma on the initial CT imaging. In contrast to type 1A, these hematomas may be more dependent on vasoreactivity and delayed changes in the blood flow related to seizures and autonomic dysregulation [18]. In our senior author’s experience, type 1B hematomas typically are identified by follow-up imaging and are not accompanied by a change in the patient’s status. The cases of DTICH occurring more than a week after trauma reported by Alvarez-Sabin et al. [5] are consistent with our experience with type 1B. However, while they did not see a significant neurological decline with type 1B, their patients did not sustain severe trauma requiring neurosurgical intervention [5]. By contrast, cases of type 1B reported by Gudeman et al. [23] and Elsner et al. [3] resulted in severe disability or death. Thus, we recommend close monitoring of patients that develop type 1B DTICH. These patients likely do not require surgical intervention but should be followed up with serial imaging, and anticoagulation should be held. While no current method for prevention exists, given the non-negligible incidence of type 1B, we recommend imaging for patients in the weeks after severe head trauma and early follow-up in the clinic.

DTICH type 2 is classified as de novo hematomas that form following decompressive craniectomy for an unrelated post-traumatic hematoma. These hematomas typically form contralateral to the initial hematoma and occur due to de-tamponading of venous oozing [19]. DTICH type 2 is not an uncommon occurrence. Yang et al. [7] reported that 7.4% of 108 cases of decompressive craniectomy developed contralateral hematoma. New hematomas can be more clinically serious and require a return to the operating room. The prevention of this phenomenon may not be possible. A strong index of suspicion is required if contralateral to the initial hematoma or imaging shows a skull fracture or pneumocephalus. If these characteristics are seen, we recommend post-operative immediate CT or intraoperative CT if possible. In addition, intraoperative ultrasound can aid management and screening for DTICH type 2. ICP monitoring is essential after surgery to identify hematoma formation. However, currently, there are no known precautionary measures or techniques for prevention. The identification of risk factors for DTICH type 2 is needed.

DTICH type 3 is a hematoma that develops in areas of non-hemorrhagic contusion after physiologic derangements, such as hypotension, rebound hypertension, hypoxia, or hypercarbia [8]. Brain contusion may cause disturbed autoregulation, vasoreactivity, and vascular permeability that, when compounded with a physiologic insult, predisposes to hemorrhage [20]. Type 3 often occurs after procedures for other non-neurosurgical traumas. Type 3 is rarer than the other types [8], likely due to modern advances in anesthesiology and critical care that prevent severe physiologic alterations. However, in our experience, type 3 does occur. We recommend appropriate ICP monitoring after head trauma, especially for those patients that undergo non-neurosurgical procedures and careful physiologic control in head trauma patients to prevent hematoma formation. In addition, Lee and Lui [8] recommended early CT imaging for patients who receive massive transfusion after severe trauma to screen for delayed hematomas. We agree with Lee and Lui [8] and advocate for follow-up imaging in all patients with head trauma who undergo non-neurosurgical procedures or receive significant volume resuscitation for blood loss or hypotension.

The main limitation of our review of the pathophysiology of DTICH is that not all studies obtained vascular imaging. While an absence of a vascular source of delayed hemorrhage was confirmed with CTA or formal angiography in some cases, we cannot rule out vascular injury as a confounder in other reports. Future investigation into the etiologies of DTICH will require angiography to identify any vascular injury. Similarly, pseudoaneurysms are potential sources of small intracerebral hematomas. Post-traumatic intracranial pseudoaneurysms are rare, accounting for less than 1% of intracerebral aneurysms [29], but they may be underreported due to a lack of angiographic imaging in many cases. In addition, there is no standard means of screening for pseudoaneurysms, and the timing of their development is not well characterized.

## Conclusions

DTICH is a recently underrecognized phenomenon, which deserves renewed interest in the field of neurotrauma, as DTICH is a common sequela of head trauma with a high associated morbidity. We propose a pathophysiologic classification system, which accounts for the distinct, recognized etiologies of DTICH, each of which requires unique management strategies. Future studies are required to continue the characterization of the pathophysiology of the DTICH types (1A, 1B, 2, and 3) and further define effective strategies for the prevention and early recognition of a developing DTICH.
